# What crystal engineering can teach us about water treatment.

**DOI:** 10.1063/4.0001086

**Published:** 2025-10-27

**Authors:** Michael A. Reynolds

**Affiliations:** Shell Catalysts and Technologies, Houston, Texas, USA

## Abstract

Acid-insoluble inorganic scales such as the sulfates of calcium, barium, and strontium are problematic contaminants that are commonly encountered in pipelines and water treatment facilities. These biominerals have low solubilities in water (Ksp < 10^-10^) and are resistant to changes in pH, temperature, and flow conditions. In addition to these issues, naturally occurring radium-226 can co-crystallize within these scales leading to radioactive hazards. Most methods for remediation involve abrasive mechanical milling techniques that can damage equipment and increase maintenance costs for well or water processing operations. A more advantageous option would be to apply chemicals that can either inhibit scale formation, or dissolve scale that has already precipitated. Yet, few viable chemical options are commercially available. This presentation will focus on an approach to studying scale formation and remediation using the concept of a lab-on-a-chip. Recent results from laboratory studies of new chemical inhibitors and dissolvers based on green chemistry motifs will be introduced. A goal of this presentation is to introduce the scientific community to these challenges and to demonstrate the power of collaborative research with university partners.

